# Shared Decision Making Interventions for Parents of Children on the Autism Spectrum: A Systematic and Scoping Review

**DOI:** 10.1007/s10597-026-01593-x

**Published:** 2026-03-02

**Authors:** Shely Khaikin, Beth Pfeiffer, Amber Pomponio Davidson, Andrea Goldstein Shipper, Stephanie Roth, Joanne Nicholson, Bryan Mccormick, Alexander G. Fiks, David S. Mandell, Yaara Zisman-Ilani

**Affiliations:** 1 Shared Decision Making Laboratory, Temple University, Pennsylvania Philadelphia, USA; 2 Barnett College of Public Health, Temple University, Pennsylvania Philadelphia, USA; 3 Cooper Medical School, Rowan University, New Jersey Camden, USA; 4 Medical Libraries, Christiana Care Health System, Delaware Wilmington, USA; 5 Heller School for Social Policy and Management, Brandeis University, Massachusetts Waltham, USA; 6 Children’s Hospital of Philadelphia, Pennsylvania Philadelphia, USA; 7 Perelman School of Medicine, University of Pennsylvania, Pennsylvania Philadelphia, USA; 8 Division of Psychology and Language Sciences, University College London, London, UK; 9 Department of Psychiatry & Behavioral Science, Lewis Katz School of Medicine, Temple University, Philadelphia, USA

**Keywords:** Autism, Shared decision making, Parent, Family

## Abstract

Parents of children on the autism spectrum face challenging treatment-related decisions, often with limited knowledge about available options. Shared Decision Making (SDM), a process where clinicians, patients, and families collaborate to make decisions based on evidence and preferences, can assist parents in navigating these choices. However, little is known about the use of SDM interventions for autism-related parental decisions. A systematic and scoping review was conducted across four databases (PubMed, Embase, Web of Science, and PsycInfo), and grey literature in two clinical trial registries. Study selection was conducted in two phases: title and abstract screening and full-text screening. From 7,610 records identified, two studies were included from Australia and Italy, describing multicomponent SDM interventions for parents of young children (< 18 years) on the autism spectrum. Both interventions demonstrated improvements in SDM-related outcomes, including parents’ knowledge of autism treatments (such as speech pathology services and Early Intensive Behavior Intervention) and parents’ involvement in treatment discussions. This review reveals a critical gap in SDM intervention research for autism parental decision-making. Despite the critical role parents play in autism treatment decisions, evidence-based SDM interventions remain scarce. This finding is significant given the well-established benefits of SDM in other healthcare populations and underscores the urgent need to develop and rigorously evaluate SDM interventions tailored to autism care contexts that support parents in making informed decisions about their children’s care.

**Systematic review registration**: A protocol was registered on PROSPERO.

**Supplementary Information:**

The online version contains supplementary material available at 10.1007/s10597-026-01593-x.

## Introduction

Parents of children on the autism spectrum must navigate difficult treatment decisions due to the heterogeneous nature of autism itself (Karst & van Hecke, 2012; Smith & Anderson, 2013). Autism spectrum disorder is characterized by considerable variability in symptom presentation, severity, and co-occurring conditions (Hirota & King, 2023). This heterogeneity creates diverse and individualized support needs across multiple domains, including social communication, behavioral regulation, sensory processing, and daily living skills (Levy et al., 2003). Furthermore, children with autism frequently experience co-occurring conditions such as anxiety, behavioral challenges, and attention-deficit/hyperactivity disorder (ADHD), adding further complexity to treatment planning (Mosner et al., 2019). Given this variability, parents are often offered numerous treatment options (Miller et al., 2012) with varying levels of evidence (Akshoomoff & Stahmer, 2006). These include pharmacological interventions to address mental and behavioral symptoms such as anxiety, aggression, and ADHD (Hyman et al., 2020b); behavioral interventions such as Applied Behavioral Analysis (ABA) and Naturalistic Developmental Behavioral Intervention (NDBI) targeting social communication, language development, and daily living skills (Hyman et al., 2020b; Mulé et al., 2022; Rosenwasser & Axelrod, 2001); and educational and therapeutic interventions including speech-language, occupational, and sensory therapies to address communication, motor skills, and sensory processing needs (Bowker et al., 2011; Case-Smith & Arbesman, 2008; Hyman et al., 2020a; Levy et al., 2016; Sandbank et al., 2020; Schaaf et al., 2012; Schaaf & Miller, 2005; Valentine et al., 2012). The breadth and diversity of these intervention approaches reflect the complex, multifaceted nature of autism and underscore the challenges parents face in determining which combination of treatments best addresses their child’s unique profile and needs.

Parents often feel lost, they do not know what to do, where and how to seek help (Zisman-Ilani, 2022), and they lack sufficient knowledge regarding the range of available treatments (Valentine, 2010) and the best treatment options for their child (Grant et al., 2016). This knowledge gap impacts their ability to make an informed decision about their child’s care (Dinora et al., 2017; Locke et al., 2020; Mire et al., 2017). In addition, not all autism treatments have strong empirical evidence supporting their efficacy (Carlon et al., 2014; Levy et al., 2003), leaving parents uncertain about which interventions are most likely to yield positive outcomes and which may have negative consequences (Miller et al., 2012). Adding to these challenges, parents also feel insufficiently involved in treatment decision-making (Levy et al., 2016), and often report that providers dismiss concerns regarding their child’s symptoms and diagnosis. This, in turn, increases parents’ distrust in the providers and can result in reluctance to initiate or adhere to the proposed treatment plan (Bent et al., 2023; Boshoff et al., 2018). Taken together, these challenges highlight the need for a structured approach that addresses both the information gap and the collaborative decision-making gap that parents experience.

Shared Decision Making (SDM) is a health communication approach specifically designed to address these challenges by improving and facilitating treatment decision-making (Charles et al., 1997, 1999; Zisman-Ilani et al., 2021). Unlike traditional independent decision-making where parents navigate treatment choices alone or receive directive recommendations from providers, SDM creates a structured collaborative process. SDM provides structured support in decision-making to patients and providers by focusing on real-time decision-making during an appointment or consultation (not before, after, or asynchronously), to create a partnership between clinicians and patients/families where both parties actively contribute their expertise to reach decisions together (Treichler et al., 2023; Treichler & Zisman-Ilani, 2022; Zisman-Ilani et al., 2025).

In the context of parental decisions, SDM actively engages parents and providers in making decisions that best align with the child’s life circumstances and values by combining clinical expertise with family knowledge and preferences. SDM is especially relevant in highly consequential and/or preference-sensitive decisions, which are decisions that significantly impact health outcomes and quality of life, and for which there is no single “right” choice but rather multiple reasonable options where the best choice depends on individual preferences (Chmielowska et al., 2023; Zisman-Ilani et al., 2017, 2021). Given the centrality of parents in autism-related decision-making (Edwards et al., 2018) and the heterogeneous nature of autism, SDM is particularly well-suited to autism management and care (Golnik et al., 2012; Hubner et al., 2016; Huffman, 2018; Levy et al., 2016; Mulé et al., 2021). Through SDM, parents can gain access to clear, evidence-based information about treatment options while providers gain insight into family values and circumstances, directly addressing the knowledge gaps, involvement needs, and trust issues described above. Parents must navigate a complex landscape of treatment options that differ widely in evidence base, accessibility, cost, and philosophical underpinnings (Golnik et al., 2012; Hubner et al., 2016). This complexity makes SDM essential to ensuring that care decisions truly align with each family’s circumstances, values, and vision for their child’s future.

Survey and interview studies among parents for children with autism demonstrated the potential value of SDM for these families. SDM was found to be associated with higher parent satisfaction and improved knowledge regarding treatments (Golnik et al., 2012; Levy et al., 2016; Lipstein et al., 2016; Mulé et al., 2021, 2022). However, it remains unclear which interventions facilitate SDM for parents of children on the autism spectrum and how effective they are. Scoping reviews are particularly suited to mapping emerging fields and identifying gaps in the literature by systematically examining the breadth and nature of available evidence (Grant & Booth, 2009; Mak & Thomas, 2022). Therefore, the purpose of this systematic and scoping review was to scope and map existing SDM interventions for parents of children on the autism spectrum, examining their characteristics, outcomes, and the extent of evidence in this field.

## Methods

### Search Strategy and Study Selection

We conducted a systematic search following the PRISMA-S extension for search reporting of scoping reviews (Grant & Booth, 2009; Mak & Thomas, 2022; Rethlefsen et al., 2021). First, biomedical librarians developed an initial PubMed (NLM) search strategy, which they tested using the PRESS Checklist for accuracy and reproducibility (McGowan et al., 2016). Second, we used this search strategy in four databases: PubMed (NLM), Embase (Elsevier), Web of Science (Clarivate Analytics) and PsycInfo (EbscoHost) using a combination of keywords and subject headings. In addition, we conducted a grey literature search in two clinical trial registries, (clinicaltrials.gov) and WHO ICTRP (https://trialsearch.who.int/).

The search was conducted from January 1, 1990 to March 15, 2022 and again from January 1, 2022 to July 7, 2023. We started the search in 1/1/1990 as accepted in previous SDM reviews (Chmielowska et al., 2023; Zisman-Ilani et al., 2017), given that the SDM literature began to emerge in the late 1990’s (Charles et al., 1997). The search syntax is available in the Appendix. Study selection was conducted in two phases. First, all studies were screened by title and abstract by two trained reviewers based on the inclusion and exclusion criteria. Disagreements were discussed and resolved, leading to strong agreement (> 90%). Second, two reviewers performed full-text screening of any potentially relevant studies. Disagreements were discussed and resolved, leading to a final sample of two included articles (Murphy et al., 2011; Strauss et al., 2015). 

## Inclusion and Exclusion Criteria

Studies were included in the review if they met all the following criteria: (1) involved an SDM intervention focusing on at least one parent of a child on the autism spectrum, (2) published from 1990 until the day of the search (July 2023) and (3) in English. We used a published SDM intervention definition (Chmielowska et al., 2023; Zisman-Ilani et al., 2017, 2021), which specifies that SDM interventions should involve at least two participants, focus on provision of information about existing treatment options and facilitate discussion of these options, and elicit patient preferences and values (Zisman-Ilani et al., 2021). Based on established classifications of SDM interventions in mental health, we classified SDM as any of the following types of interventions: decision aids, multicomponent interventions including a decision aid, multicomponent interventions not involving a decision aid, and shared care planning and preference elicitation interventions (Chmielowska et al., 2023; Zisman-Ilani et al., 2017). Studies were excluded if they were (1) published before 1990, (2) not in English, (3) not including a parent of a child on the autism spectrum, and (4) did not utilize an SDM intervention (e.g., studies describing SDM survey level or SDM qualitative interviews).

## Data Extraction

Characteristics of each study (e.g., country of origin and demographic characteristics), experimental intervention (e.g., objective, setting, type of intervention, study arm), and methods (e.g., study design and measures) were extracted (see Table 1a and b).

**Table 1:** Characteristics of the included studies

Title	Authors	Country	Year	Aim	Study design	Setting	Measures	Intervention type	Study arm		
Supporting parents of children with autism spectrum disorders to become informed consumers of evidence on speech pathology practice	Annemarie Murphy, David Trembath, Joanne Arciuli & Jacqueline M. Roberts	Australia	2011	To examine the effectiveness of an intervention program to help parents of children on the autism spectrum in making informed decisions regarding evidence-based speech pathology services.	Repeated measures within-group pre-post	A community program providing early intervention and support for children on the autism spectrum and their parents.	Self-report: a tailored a questionnaire consisting of yes/no, Likert scale ratings, and open-ended questions.	Multicomponent SDM intervention involving DA (information sheets).	Intervention arm was divided into two: one group received the information sheets followed by the workshop while the other group underwent the workshop first followed by the information sheets.		
Promoting Shared Decision Making to strengthen outcome of young children with Autism Spectrum Disorders: The role of staff competence	Kristin Strauss, Arianna Benvenuto, Barbara Battan, Martina Siracusano, Monica Terribili, Paolo Curatolo, Leonardo Fava	Italy	2015	To compare the effects of various models of parent involvement in Early Intensive Behavioral Interventions (EIBI) for young children on the autism spectrum and to explore the potential correlation of incorporated SDM staff training with positive child developmental progress and parent outcome.	Quasi-experimental design	Autism treatment center and a community setting.	Self-report: The revised version of the Observing Patient Involvement Scale (OPTION), Autism Diagnostic Observation Schedule and (ADOS-2), Psychoeducational Profile-Third Edition (PEP-3) Vineland Adaptive Behavior Scales-Second Edition (VABS), Parental Stress Index-Short Form (PSI-SF), Early Intervention Parenting Self-Efficacy Scale (EIPSES), Belief in Intensive Behavioral Intervention (BIBI) scale	Multicomponent SDM intervention without DA.	Intervention arm was divided in two: Parent Inclusion in Treatment Delivery Only (PI-DO) and Parent Involvement accompanied by SDM Staff Training (PI-SDM). A third study group was treatment as usual (TAU)		
b. Characteristics of the Included Studies											
Title	Participants	Sample size	Parent characteristics (relation to child)	Parent age	Parent sex	Race	Child diagnosis	Child sex	Child age	Age child diagnosed	Provider characteristics
Supporting parents of children with autism spectrum disorders to become informed consumers of evidence on speech pathology practice	Parents	n = 28 **Only 12 completed the post-test questionnaire.	Mothers = 26Fathers = 2	no mention	Females = 26Males = 2	no mention	Autism	no mention	Range: 29 to 60 months (*M* = 43 months)	Between 1 and 24 months (*M* = 13 months)	no mention
Promoting Shared Decision Making to strengthen outcome of young children with Autism Spectrum Disorders: The role of staff competence	Children with ASD and their parents	n = 25	Only maternal data (n = 25) is reported due to the lack of consistent data from fathers for all children.	no mention	Females = 25	no mention	A diagnosis of ASDs, including Autistic Disorders (AD) and Pervasive Development Disorders-Not Otherwise Specified (PDD-NOS).	Males = 23Females = 3	Aging from 34 to 92 months with a mean age of 51.41 ± 13.62 months	no mention	Staff that had approximately five years of experience as a therapist, but not more than one or two years of experience with independent case and family management.

## Risk of Bias and Study Quality Assessment

Risk of bias for each study was independently evaluated by a pair of reviewers using the Cochrane Collaboration Risk-of-Bias tool (Higgins et al., 2016; Sterne et al., 2019). Discrepancies were resolved by discussion until consensus was reached.

## Results

### Search Results

**Fig. 1:**
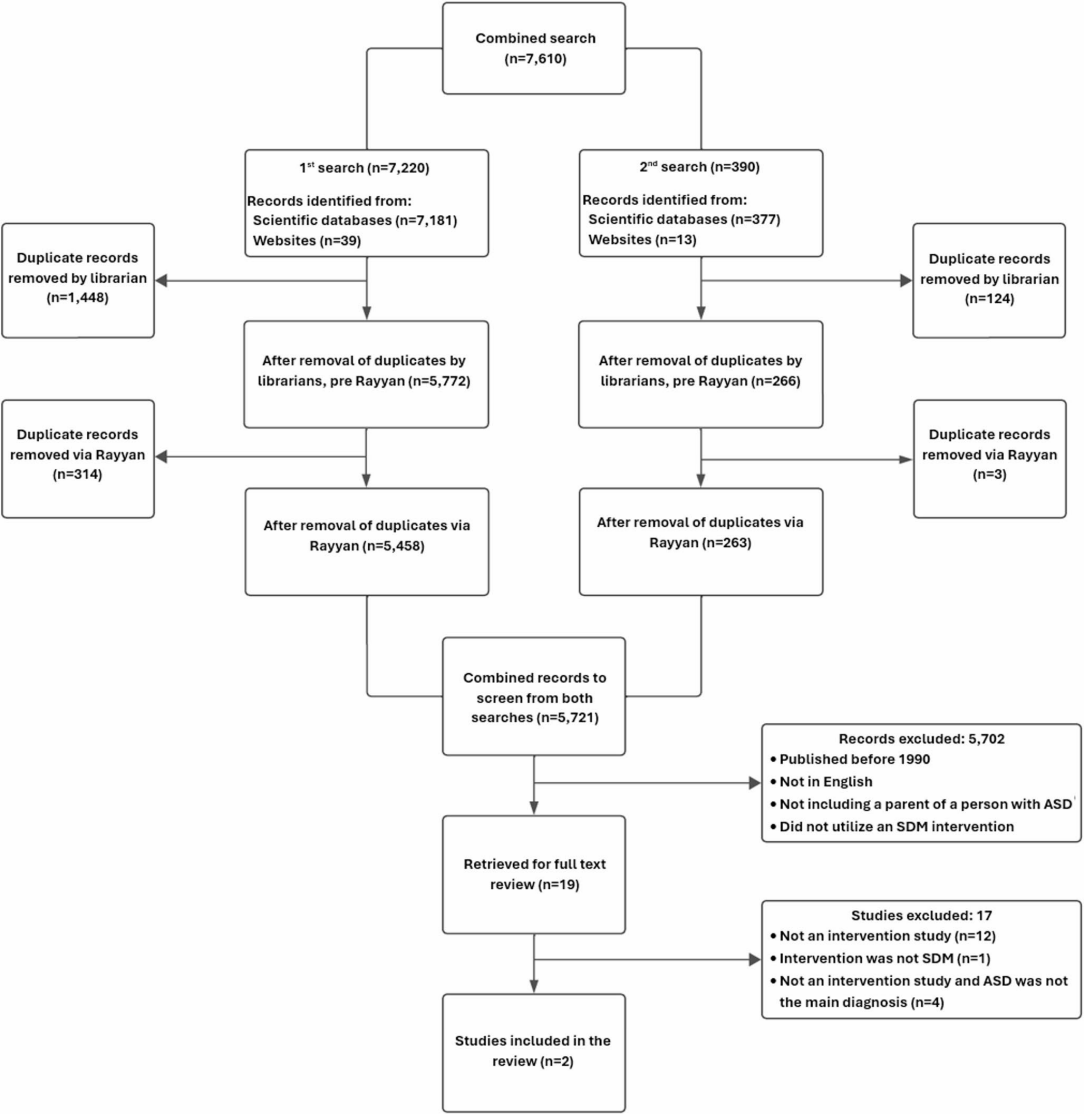
A PRISMA flow diagram

Figure 1 (see Appendix) describes the flow of information and selection process through the different review phases. The systematic database search (before removal of duplicates) was combined of two searches (the second was an updated search) and resulted in a total of 7,610 records (including a total of 52 grey-literature records). The first search (conducted on 3/15/2022) yielded 7,220 records, of which, 1,448 duplicate records were found and omitted by the librarian using EndNote 20 duplicate identification strategy, resulting in 5,772 records. These records were imported to Rayyan, a systematic review collaborative platform, which automatically detected and removed additional 33 duplicate records. A manual check for duplicates in Rayyan identified additional 281 records to remove, resulting in a total of 5,458 total records ready to screen from search 1. The second search (conducted on 7/7/2023) yielded 390 records, of which, 124 duplicate records were found and omitted by the librarian using EndNote 20, resulting in 266 records. These records were imported to Rayyan, and a manual check for duplicates in Rayyan identified three more records which were removed, resulting in a total of 263 ready to screen records in search 2. The combined records to screen from both searches was 5,721 (Fig. 1). Of these, 19 records were retrieved for full-text review and two studies were included in the final sample (Murphy et al., 2011; Strauss et al., 2015). Seventeen studies were excluded (Fig. 1) because their design was not an intervention study (*n* = 12), the design was not an intervention and autism was not the main diagnosis of the child (*n* = 4), or the included intervention did not meet the SDM definition (*n* = 1).

## Risk of Bias and Study Quality

The included studies represent pioneering efforts, as they tested SDM interventions during the early stages of SDM development in behavioral health and autism care. Both interventions were conducted between 2011 and 2015, shortly after SDM approaches began expanding into mental health settings in the late 2000s. Given that these interventions represent early SDM efforts in the field, several methodological issues related to risk of bias and quality were identified. According to the Cochrane Collaboration Risk-of-Bias tool, both studies had a high risk of bias and low methodological quality, due to limited reporting on the randomization process (Murphy et al., 2011), deviations from the intended interventions (Murphy et al., 2011; Strauss et al., 2015), and missing background and outcome data (Murphy et al., 2011). For example, both studies lacked information on participants’ socioeconomic status and educational attainment (Strauss et al., 2015). In Murphy et al., only 43% (12/28) of participants completed the post-test questionnaire, and Strauss et al. had a small sample size.

## Studies Characteristics

The study by Murphy et al. was conducted in Australia and the study by Strauss et al. was conducted in Italy. Murphy et al. reported data from both mothers and fathers, while Strauss et al. reported only maternal data. Both studies included parents of young children on the autism spectrum, aged 29 to 60 months (Murphy et al., 2011) and 34 to 92 months (Strauss et al., 2015). Tables 1a and b describe the characteristics of the included studies (Murphy et al., 2011; Strauss et al., 2015).

### Study Design

Murphy et al. used a repeated measures within-group design (pre/post) using surveys and open-ended questions. One group of parents of children on the autism spectrum (*n* = 28), who was engaged in an early intervention and support group program, was assessed at baseline and post intervention (Murphy et al., 2011). Strauss et al. conducted a quasi-experimental between-groups design. Parents and children in an Early Intensive Behavior Intervention (EIBI) program were randomly allocated to two groups: Parent Inclusion in Treatment Delivery Only (PI-DO) and Parent Involvement accompanied by SDM Staff Training (PI-SDM). Participants were blinded to group allocation. A third study group consisting only of children receiving non-experimental governmental treatment as usual (TAU) served as a pair matched control (*n* = 8) for children only. Participants in all groups underwent a follow-up one year after their initial evaluation, which was aligned with 9 months of intervention.

### Description of Interventions

Murphy et al. examined the effects of a multicomponent SDM intervention involving a decision aid and an interactive workshop designed to help parents make informed decisions about evidence-based speech pathology services for their child. The intervention consisted of printed information sheets provided to parents (a “decision aid”) and a workshop. Participants received the intervention in two formats: nine participants were given the information sheets followed by the workshop, and 19 received the workshop first followed by the information sheets. The information sheets aimed to aid parents with knowledge on evidence-based treatments as a guide for decision-making, offered evaluations of the levels of evidence of treatments available for children on the autism spectrum, and provided a set of questions designed to assist parents in asking professionals about the efficacy of various interventions. The sheets were distributed to the participants and were made accessible online during the study period (as reported by the authors). Similar information covered in the information sheets was presented in the workshop with participants having the opportunity to bring up personal experiences, ask questions, and comment on the workshop’s content. To aid note taking, participants were provided with a hard copy of the slides to take home.

Strauss et al. examined the impact of a multicomponent SDM intervention, delivered without a decision aid, on child and parent outcomes in EIBI when parents were involved. EIBI, which is based on developmental and behavioral approaches, is commonly used as a treatment for autism(Reichow et al., 2018; Strauss et al., 2015). EIBI treatment outcomes were compared to TAU. Children in both experimental groups (PI-SDM and PI-DO) received 15 weekly hours of EIBI, whereas children in the TAU matched control group received five weekly hours of non-EIBI treatment. Child participants were evaluated post-intervention for autism severity, adaptive behaviors, and developmental level. Parent participants were evaluated for their perceived involvement, beliefs in EIBI efficacy, self-efficacy, and stress (Strauss et al., 2015). Participants in the PI-SDM group followed a treatment protocol based on the child’s strengths and weaknesses and the parents’ priorities. The SDM elements included a deliberate focus on parents’ goals, needs, concerns, and priorities for their child’s treatment. The relationship between treatment priorities and children’s level of functioning was assessed using a survey developed to identify parents’ priorities (Pituch et al., 2011). To ensure integration of the intervention into family’s everyday activities, monthly meetings between supervisors and families were conducted. Moreover, community sessions, such as going to the grocery or playground with the child, were incorporated to support family and child participation in meaningful activities. Participants in the PI-DO group also followed a treatment protocol based on the child’s strengths and weaknesses and parents received theoretical and practical training as well as personal weekly supervision meetings; however, no emphasis was placed on parents’ goals, needs, concerns, and treatment priorities.

### Delivery of Interventions

Both interventions were delivered face-to-face (Murphy et al., 2011; Strauss et al., 2015). The workshop part in Murphy et al. was delivered by two experienced clinicians and researchers in the field of autism and lasted 1.5 h. In Strauss et al., experienced EIBI therapists delivered the intervention. SDM training for the interventionists of the PI-SDM group was provided by neuropsychiatrists, child psychologists, and the program director, and was held bimonthly. The goal of the training and coaching sessions was to increase interventionists’ competencies, enhance their ability to prepare parents for active involvement in decision-making, and integrate parent input into treatment planning.

### Measures

To examine changes in participants’ self-reported knowledge of evidence-based practice (EBP) speech pathology services and their confidence in discussing these services with their children’s speech pathologists, Murphy et al. tailored a questionnaire consisting of yes/no, Likert scale ratings, and open-ended questions (Murphy et al., 2011). The questionnaire gathered qualitative information about parents’ perspectives on and experiences with accessing EBP speech pathology services (Murphy et al., 2011). Murphy et al. did not administer SDM scales. Strauss et al. used the revised version of the Observing Patient Involvement Scale (OPTION), an SDM acceptable measure (Fattori et al., 2023; Perestelo-Perez et al., 2017) to evaluate the level and quality of the SDM process. Additional child and parent psychological evaluations, including cognitive and adaptive measures, were administered in this study. Child measures consisted of a psychological evaluation which included the Autism Diagnostic Observation Schedule (ADOS-2), the Psychoeducational Profile-Third Edition (PEP-3), and the Vineland Adaptive Behavior Scales-Second Edition (VABS). Parents completed self-report measurements, including the Parental Stress Index-Short Form (PSI-SF), the Early Intervention Parenting Self-Efficacy Scale (EIPSES), and the Belief in Intensive Behavioral Intervention (BIBI) scale.

### Outcomes of Interventions

Murphy et al. reported that, post-intervention, parents experienced an improvement in their understanding of the range of treatments available for their child (pre 3.89 ± 0.78, post 4.44 ± 0.53, *p* = .025). They expressed increased awareness of the research evidence supporting the treatments being used (pre 2.89 ± 0.93, post 4.11 ± 0.33, *p* = .018) and felt more knowledgeable about what questions to ask their provider regarding their child’s treatment (pre 3.00 ± 0.87, post 4.00 ± 0.71, *p* = .014). No significant changes were found in parents’ comfort asking questions (pre 4.00 ± 1.00, post 4.11 ± 0.78, *p* = .655) and in their knowledge of what ‘evidence-based practice’ means (pre 3.89 ± 1.05, post 4.11 ± 0.60, *p* = .527). Parents differed in their preferred method of information delivery. Among the eight parents who responded to the method preference question, five preferred the workshop (62.5%) and three (37.5%) preferred the information sheets. In open-ended responses, parents suggested that, to better facilitate informed decision-making, professionals should use clearer language when explaining post-diagnosis actions, foster a professional culture that promotes greater acceptance of parents, and provide more consistent information across professionals.

Strauss et al. reported significant improvements in SDM outcome measures in the PI-SDM group, (OPTION competence (pre 52.78 ± 2.55, post 74.31 ± 6.16, *p* = .0001) and OPTION perception (pre 80.78 ± 15.44, post 93.06 ± 7.86, *p* = .031). In the PI-PO group, SDM outcomes were not significant for OPTION competence (pre 51.52 ± 3.08, post 63.67 ± 6.56, *p* = .392) and OPTION perception (pre 88.69 ± 14.42, post 97.91 ± 2.94, *p* = .178). Parents in the PI-SDM group also reported increased beliefs in the beneficial effects of EIBI on their child’s development (BIBI my child: pre 21.00 ± 2.39, post 25.50 ± 2.27, *p* = .040), as well as enhance self-efficacy related to EIBI parenting competence (EIPSES competence: pre 41.67 ± 2.55, post 46.00 ± 4.12, *p* = .011). In the PI-PO group, no significant improvements in BIBI my child (pre 22.00 ± 3.50, post 22.33 ± 4.53, *p* = .754) was measured (Strauss et al., 2015). Additionally, as supervisors’ SDM competencies significantly increased over time in the PI-SDM intervention group (*p* ≤ .001), parental involvement gradually increased (*p* ≤ .01). Parents in the PI-PO group demonstrated higher involvement although limited improvements were reported in supervisors’ SDM competence. Additionally, in the PI-PO group, a significant increase in parental perceptions of parenting competence (pre 38.25 ± 6.73, post 44.67 ± 6.31, *p* = .011) was accompanied by a significant decrease in EIBI outcome expectations (pre 31.00 ± 4.95, post 23.00 ± 5.93, *p* = .005), leading to increased reliance on staff competence. This pattern suggests that as parents’ expectations of EIBI decreased, they became more dependent on staff’s skills. As for clinical outcomes, children in both the PI-SDM and PI-PO groups showed significant clinical improvements on standardized measures, including VABS, PEP communication, and PEP motor. Results from individual clinical outcomes further indicated that children of parents in the PI-SDM group showed significant improvement on the ADOS-2. No significant improvements in autism severity and developmental skills were found in the PI-PO and TAU group.

## Discussion

Parents have significant decision-making responsibility in the treatment of their autistic children. SDM interventions assist with decision-making by enhancing decision-makers’ self-efficacy, knowledge, and health literacy regarding treatment options (Zisman-Ilani et al., 2017, 2021). SDM interventions also aim to increase treatment engagement, which in turn can support improved clinical outcomes (Zisman-Ilani et al., 2017, 2021). Although parents of children on the autism spectrum may benefit from SDM interventions, this systematic and scoping review highlights a significant gap in both clinical practice and research. Only two evidence-based SDM interventions were identified that were specifically designed for parents of young children on the autism spectrum. This finding is particularly notable because our search was not limited to young children, indicating an absence of SDM interventions for parents of older children, teens, and young adults. In alignment with SDM theory and practice, these two interventions were designed to help parents become informed consumers of evidence-based speech pathology interventions (Murphy et al., 2011) and to support parent engagement during EIBI for young children (Strauss et al., 2015).

The interventions identified in our review showcase the potential of SDM in autism. For example, the intervention administered by Murphy et al. led to improvements in parents’ understanding of the range of evidence-based speech pathology practices available to their child. This is critically important, as parents of children on the autism spectrum are often unfamiliar with this information, which can lead to parents’ disengagement from treatments (MacKintosh et al., 2012; Miller et al., 2012; Moon, 2010; Wilson et al., 2018). Murphy’s SDM intervention also increased parents’ awareness of the research evidence supporting the treatments being used with their child, aligning with cumulative findings from family treatment research demonstrating that parental engagement has a critical influence on intervention outcomes for children on the autism spectrum (Hock et al., 2015; King et al., 2022). Strauss et al. reported that parents’ involvement in EIBI and their beliefs regarding the effectiveness of the treatment increased after communicating with supervisors trained in SDM. In Strauss et al., children whose supervisors were proficient in SDM showed improved outcomes on psychological evaluations, whereas children whose supervisors had not received SDM training did not show significant improvements in clinical outcomes. This underscores the importance of SDM training and support for providers.

The two included studies also demonstrate significant benefits for the well-being of parents of young children on the autism spectrum and for the children themselves. Compared to parents of neurotypical children, parents of children on the autism spectrum, experience numerous stressors and burdens (e.g., economic hardships, daily hassles related to child behavior, limited social support and access to services, and anxiety about the child’s future) (Costa et al., 2017; Curley et al., 2023; Gur & Rimmerman, 2021; Krakovich et al., 2016). Therefore, identifying strategies that help these parents reduce stress and mitigate the consequences of their adverse experiences is essential. As demonstrated in both studies (Murphy et al., 2011; Strauss et al., 2015), routinely and systematically implementing SDM in autism services may help parents improve their self-efficacy and knowledge of treatment options. Although not directly tested in these studies, consistent evidence from other SDM studies(Chmielowska et al., 2023; Zisman-Ilani et al., 2017) indicated that SDM can also strengthen the therapeutic alliance and increase trust between parents and providers, enhancing service engagement and functional outcomes. The SDM intervention also helped parents prioritize the most valued and relevant interventions for their child and situation (Strauss et al., 2015). For children on the autism spectrum, participation in an SDM autism parental intervention was associated with clinical improvements on standard scores in most child outcomes (Strauss et al., 2015).

Despite the positive impact demonstrated by the two intervention studies, neither focused on parental decision-making about commonly prescribed pharmacological treatments for children on the autism spectrum. While debate continues in the field regarding the appropriateness of pharmacological treatment for children with autism (Huffman et al., 2011), prescribing practices persist, and SDM interventions could help parents navigate these complex medication decisions. The current lack of SDM support around psychiatric medications for children with autism leaves parents inadequately supported, lacking thorough understanding of the risks, benefits, and evidence for treatment options (Levy et al., 2003). Furthermore, the included studies were published over a decade ago. During the past decade, there was a surge in SDM interventions across clinical settings and conditions, particularly in mental health (Treichler et al., 2023; Zhao et al., 2024; Zisman-Ilani et al., 2024; Zisman-Ilani, Hayes et al., 2023a; Zisman-Ilani, Li et al., 2023b; Zisman-Ilani & Byrne, 2022). The absence of other SDM interventions designed for parents of children on the autism spectrum is noteworthy and highlights a blind spot within the SDM field, where triadic or multi-dyadic decision-making situations, or surrogate decision-making, has not received adequate attention. Moreover, over the last two decades, multiple new therapies have emerged, adding to the complexity of parental decision-making and highlighting the need to develop additional SDM interventions, particularly those tailored for parents. We also found no SDM interventions targeting parents of adolescents or young adults. Each of these age groups experiences unique needs and challenges, suggesting that existing SDM interventions for early childhood may not translate effectively for later developmental periods (Burke et al., 2024; Narendorf et al., 2011; Smith & Anderson, 2013; Steinberg et al., 2024). Taken together, the findings of this systematic and scoping review clearly point to the need for developing and testing SDM interventions that support parents of individuals on the autism spectrum across the lifespan.

The findings of this review underscore the need for rigorous development of additional SDM interventions for parents of children on the autism spectrum, who serve as the primary caregivers and decision-makers, particularly for younger children. Overall, the findings from the included studies illustrate SDM’s contributions to parents’ decision-making, engagement, self-efficacy, treatment literacy, and demonstrate that children also benefit through their parents’ increased involvement and knowledge.

Based on the two interventions identified, providers can begin implementing SDM principles in autism care settings. Providers can: (1) develop or adapt decision aids that present evidence-based treatment options in accessible formats, including evaluation of evidence levels and questions parents can ask about treatment efficacy; (2) incorporate interactive educational workshops that allow parents to discuss personal experiences and ask questions in a supportive group setting; (3) assess and integrate parents’ goals, needs, concerns, and priorities into treatment planning; (4) hold regular meetings with families to ensure interventions are integrated into everyday family activities and routines; and (5) include community-based sessions that support meaningful family participation in real-world contexts. These practical strategies, drawn from the existing evidence, can be adapted across different autism treatment settings while research continues to develop.

However, clinicians should recognize that the current evidence base is limited to young children, and future SDM interventions should be developed and tailored to specific age groups, autism diagnoses, and individual needs. Additional research should examine parents’ information-seeking behaviors and existing sources of support, as well as assess as the potential impact of decision quality on child and family outcomes. This work represents an important step toward ensuring that all families affected by autism have access to evidence-based decision-making support tailored to their unique circumstances.

## Supplementary Information

Below is the link to the electronic supplementary material.

Supplementary Material 1 (PDF 171 KB)

## Data Availability

No datasets were generated or analysed during the current study.

## Abbreviations

SDM: Shared decision making
ADHD: Attention-deficit/hyperactivity disorder
ABA: Applied Behavioral Analysis
NDBI: Naturalistic Developmental Behavioral Intervention
EBP: Evidence- based practice
EIBI: Early Intensive Behavior Intervention
PI-DO: Parent Inclusion in Treatment Delivery Only
PI-SDM: Parent Involvement accompanied by SDM Staff Training
TAU: Treatment as usual
OPTION: Observing Patient Involvement Scale
ADOS-2: Autism Diagnostic Observation Schedule
PEP-3: Psychoeducational Profile-Third Edition (PEP-3)
VABS: Vineland Adaptive Behavior Scales-Second Edition
PSI-SF: Parental Stress Index-Short Form
EIPSES: Early Intervention Parenting Self-Efficacy Scale
BIBI: Belief in Intensive Behavioral Intervention
