# Supplementary material for: Shared Decision Making Interventions for Parents of Children on the Autism Spectrum: A Systematic and Scoping Review
Source: Community Ment Health J. 2026 Mar 2;62(6):1054–66. doi: 10.1007/s10597-026-01593-x (PMC13451329; doi:10.1007/s10597-026-01593-x)
Supplement: Supplementary file 1 [file 10597_2026_1593_MOESM1_ESM.pdf]

Search Methods (first search)

Prepared by removed for a blind review

3/11/2022; 3/15/2022 Revised

PubMed (NLM) *from 01/01/1990 to 3/4/2022* (1,441 Results)

("Neurodevelopmental Disorders"[Mesh] OR "neurodevelopment disorder*"[tiab] OR "developmental delay*"[tiab] OR "Communication Disorders"[Mesh] OR "communication disorder*"[tiab] OR "developmental communication disorder*"[tiab] OR "childhood communication disorder*"[tiab] OR "communicative dysfunction*"[tiab] OR "communication disability*"[tiab] OR "Language Disorders"[Mesh] OR "Acquired Language Disorder*"[tiab] OR "language disorder*"[tiab] OR "Speech Sound Disorder"[Mesh] OR "speech sound disorder*"[tiab] OR "phonological disorder*"[tiab] OR "Speech-Sound Disorder, hereditary"[Supplementary Concept] OR "speech sound disorder*"[tiab] OR "Childhood-Onset Fluency Disorder"[Mesh] OR "childhood onset fluency disorder*"[tiab] OR "fluency disorder*"[tiab] OR "Stuttering"[Mesh] OR stutter*[tiab] OR "Social Communication Disorder"[Mesh] OR "Social Communication Disorder*"[tiab] OR "Pragmatic Communication Disorder*"[tiab] OR 'autism'/exp OR "autism spectrum disorder*"[tiab] OR autis*[tiab] OR "Autistic Disorder"[Mesh] OR "Attention Deficit Disorder with Hyperactivity"[Mesh] OR "Attention Deficit Disorder*"[tiab] OR hyperactivit*[tiab] OR "ADHD"[tiab] OR "ADDH"[tiab] OR "Attention Deficit Hyperactivity Disorder*"[tiab] OR "hyperkinetic syndrom*"[tiab] OR "Specific Learning Disorder"[Mesh] OR "specific learning disorder*"[tiab] OR "Motor Disorders"[Mesh] OR "motor disorder*"[tiab] OR "Motor Skills Disorders"[Mesh] OR "motor skills disorder*"[tiab] OR "developmental coordination disorder*"[tiab] OR "Stereotypic Movement Disorder"[Mesh] OR "stereotypic movement disorder*"[tiab] OR "Movement Disorder*" OR "head bang*"[tiab] OR "Tic Disorders"[Mesh] OR "tic disorder*"[tiab] OR "chronic motor tic disorder*"[tiab] OR "vocal tic disorder*"[tiab] OR "chronic tic disorder*"[tiab] OR "Intellectual disabil*"[tiab] OR "learning disabil*"[tiab] OR autism*[tiab] OR "developmental disabil*"[tiab] OR "Intellectual Disability"[MeSH] OR "Learning Disabilities"[Mesh] OR "IDD"[tiab] OR "intellectual developmental disabil*"[tiab] OR "IDD MH"[tiab] OR "intellectual disabilit*"[tiab] OR "Developmental Disabilities"[Mesh] OR "developmental disabilit*"[tiab] OR "developmental disorder*"[tiab]) AND (("Decision Making, Shared"[Mesh] OR "shared decision mak*"[tiab] OR "decision aid*"[tiab] OR "decision support*"[tiab] OR "decision making*"[tiab] OR "decision making intervention*"[tiab] OR "decision making tool*"[tiab] OR "SDM"[tiab] OR "shared clinical decision*"[tiab] OR "shared medical decision*"[tiab] OR (patient*[tiab] AND empower*[tiab]) OR "Decision making"[MeSH] OR "decision support techniques"[Mesh] OR "patient participation"[MeSH] OR "patient participat*"[tiab] OR "decision support systems,

clinical"[Mesh] OR "decision trees"[MeSH] OR "Choice Behavior"[Mesh] OR "Decision Making"[Mesh] OR "decision tree*"[tiab] OR "choice behavior*"[tiab] OR "Decision Making, Shared"[Mesh] OR "Shared medical decision*"[tiab] OR "Shared treatment decision*"[tiab] OR "Shared clinical decision*"[tiab]) OR (((decision*[tiab] or decid*[tiab]) AND (support*[tiab] OR "aid"[tiab] OR tool*[tiab] OR instrument*[tiab] OR technique*[tiab] OR system*[tiab] OR program*[tiab] OR process*[tiab] OR method*[tiab] OR intervention*[tiab] OR material*[tiab])))) AND ("Parents"[MeSH] OR parent*[tiab])
Filters: from 1990/1/1 - 3000/12/12

Embase (Elsevier) *from 01/01/1990 to 3/4/2022* (3,684 Results)

('mental disease'/exp OR 'mental diseas*':ti,ab OR 'neurodevelopment disorder*':ti,ab OR 'developmental delay*':ti,ab OR 'communication disorder*':ti,ab OR 'developmental communication disorder*':ti,ab OR 'childhood communication disorder*':ti,ab OR 'communicative dysfunction*':ti,ab OR 'communication disability*':ti,ab OR 'language disability'/exp OR 'language disabil*':ti,ab OR 'acquired language disorder*':ti,ab OR 'language disorder*':ti,ab OR 'speech sound disorder'/exp OR 'speech sound disorder' OR 'phonological disorder*':ti,ab OR 'speech sound disorder*':ti,ab OR 'fluency disorder'/exp OR 'childhood onset fluency disorder*':ti,ab OR 'fluency disorder*':ti,ab OR 'stuttering'/exp OR stutter*:ti,ab OR 'communication disorder'/exp OR 'social communication disorder*':ti,ab OR 'pragmatic communication disorder*':ti,ab OR 'autism'/exp OR 'autistic spectrum disorder*':ti,ab OR 'autism spectrum disorder*':ti,ab OR autis*:ti,ab OR 'attention deficit hyperactivity disorder'/exp OR 'attention deficit disorder*':ti,ab OR hyperactivit*:ti,ab OR 'adhd':ti,ab OR 'addh':ti,ab OR 'attention deficit hyperactivity disorder*':ti,ab OR 'hyperkinetic syndrom*':ti,ab OR 'learning disord*':ti,ab OR 'specific learning disorder*':ti,ab OR 'motor disorder*':ti,ab OR 'psychomotor disorder'/exp OR 'psychomotor disorder':ti,ab OR 'motor skills disorder*':ti,ab OR 'developmental coordination disorder*':ti,ab OR 'motor dysfunction'/exp OR 'motor dysfunction':ti,ab OR 'stereotypic movement disorder*':ti,ab OR 'movement disorder*':ti,ab OR 'head bang*':ti,ab OR 'tic'/exp OR 'tic*':ti,ab OR 'tic disorder*':ti,ab OR 'chronic motor tic disorder*':ti,ab OR 'vocal tic disorder*':ti,ab OR 'chronic tic disorder*':ti,ab OR 'intellectual disabil*':ti,ab OR 'learning disabil*':ti,ab OR autism*:ti,ab OR 'developmental disabil*':ti,ab OR 'intellectual impairment'/exp OR 'intellectual impairment':ti,ab OR 'learning disorder'/exp OR 'learning disorder':ti,ab OR 'idd':ti,ab OR 'intellectual developmental disabil*':ti,ab OR 'idd mh':ti,ab OR 'intellectual disabilit*':ti,ab OR 'developmental disorder'/exp OR 'developmental disabilit*':ti,ab OR 'developmental disorder*':ti,ab) AND ('shared decision making'/exp OR 'shared decision mak*':ti,ab OR 'decision aid*':ti,ab OR 'decision support*':ti,ab OR 'decision making*':ti,ab OR 'decision making intervention*':ti,ab OR 'decision making tool*':ti,ab OR 'sdm':ti,ab OR (patient*:ti,ab

AND empower*:ti,ab) OR 'decision making'/exp OR 'decision support system'/exp OR 'patient participation'/exp OR 'patient participat*':ti,ab OR 'clinical decision support system'/exp OR 'decision tree'/exp OR 'decision tree*':ti,ab OR 'choice behavior*':ti,ab OR 'shared medical decision*':ti,ab OR 'shared treatment decision*':ti,ab OR 'shared clinical decision*':ti,ab OR ((decision* NEAR/3 support):ti,ab) OR ((decision* NEAR/3 aid*):ti,ab) OR ((decision* NEAR/3 tool*):ti,ab) OR ((decision* NEAR/3 instrument*):ti,ab) OR ((decision* NEAR/3 technique*):ti,ab) OR ((decision* NEAR/3 system*):ti,ab) OR ((decision* NEAR/3 program*):ti,ab) OR ((decision* NEAR/3 process*):ti,ab) OR ((decision* NEAR/3 method*):ti,ab) OR ((decid* NEAR/3 support):ti,ab) OR ((decid* NEAR/3 aid*):ti,ab) OR ((decid* NEAR/3 tool*):ti,ab) OR ((decid* NEAR/3 instrument*):ti,ab) OR ((decid* NEAR/3 technique*):ti,ab) OR ((decid* NEAR/3 system*):ti,ab) OR ((decid* NEAR/3 program*):ti,ab) OR ((decid* NEAR/3 process*):ti,ab) OR ((decid* NEAR/3 method*):ti,ab) OR ((decid* NEAR/3 intervention*):ti,ab) OR ((decid* NEAR/3 material*):ti,ab)) AND ('parent'/exp OR parent*:ti,ab) AND [01-01-1990]/sd NOT [04-04-2022]/sd

PsycInfo (EbscoHost) *from 01/01/1990 to 3/4/2022* (1,231 Results)

((DE "Neurodevelopmental Disorders" OR DE "Attention Deficit Disorder" OR DE "Autism Spectrum Disorders" OR DE "Developmental Disabilities" OR DE "Disruptive Behavior Disorders" OR DE "Emotional and Behavioral Disorders" OR DE "Intellectual Development Disorder" OR DE "Learning Disorders") OR (TI "neurodevelopment disorder*" OR AB "neurodevelopment disorder*") OR (TI "developmental delay*" OR AB "developmental delay*") OR (DE "Communication Disorders" OR DE "Hearing Disorders" OR DE "Language Disorders") OR (TI "communication disorder*" OR AB "communication disorder*") OR (TI "developmental communication disorder*" OR AB "developmental communication disorder*") OR (TI "childhood communication disorder*" OR AB "childhood communication disorder*") OR (TI "communicative dysfunction*" OR AB "communicative dysfunction*") OR (TI "communication disability*" OR AB "communication disability*") OR (TI "Acquired Language Disorder*" OR AB "Acquired Language Disorder*") OR (TI "language disorder*" OR AB "language disorder*") OR (DE "Speech Disorders" OR DE "Aphasia" OR DE "Articulation Disorders" OR DE "Dysphonia" OR DE "Echolalia" OR DE "Mutism" OR DE "Stuttering") OR (TI "speech sound disorder*" OR AB "speech sound disorder*") OR (TI "phonological disorder*" OR AB "phonological disorder*") OR MW "Speech-Sound Disorder, hereditary" OR (TI "speech sound disorder*" OR AB "speech sound disorder*") OR (TI "childhood onset fluency disorder*" OR AB "childhood onset fluency disorder*") OR (TI "fluency disorder*" OR AB "fluency disorder*") OR (TI stutter* OR AB stutter*) OR (TI "Social Communication Disorder*" OR AB "Social Communication Disorder*") OR (TI "Pragmatic Communication Disorder*" OR AB "Pragmatic Communication Disorder*") OR

(DE "Autism Spectrum Disorders" OR DE "Autistic Traits") OR (TI "autism spectrum disorder*" OR AB "autism spectrum disorder*") OR (TI autis* OR AB autis*) OR (DE "Attention Deficit Disorder" OR DE "Attention Deficit Disorder with Hyperactivity") OR (TI "Attention Deficit Disorder*" OR AB "Attention Deficit Disorder*") OR (TI hyperactivit* OR AB hyperactivit*) OR (TI ADHD OR AB ADHD) OR (TI ADDH OR AB ADDH) OR (TI "Attention Deficit Hyperactivity Disorder*" OR AB "Attention Deficit Hyperactivity Disorder*") OR (TI "hyperkinetic syndrom*" OR AB "hyperkinetic syndrom*") OR (TI "specific learning disorder*" OR AB "specific learning disorder*") OR (DE "Nervous System Disorders" OR DE "Autonomic Nervous System Disorders" OR DE "Central Nervous System Disorders") OR (TI "motor disorder*" OR AB "motor disorder*") OR (TI "motor skills disorder*" OR AB "motor skills disorder*") OR (DE "Movement Disorders" OR DE "Alien Limb Syndrome" OR DE "Apraxia" OR DE "Ataxia" OR DE "Athetosis" OR DE "Catalepsy" OR DE "Cataplexy" OR DE "Chorea" OR DE "Dyskinesia" OR DE "Dyspraxia" OR DE "Myasthenia Gravis" OR DE "Paralysis" OR DE "Spasms" OR DE "Tics" OR DE "Torticollis" OR DE "Tremor") OR (TI "developmental coordination disorder*" OR AB "developmental coordination disorder*") OR (TI "stereotypic movement disorder*" OR AB "stereotypic movement disorder*") OR "Movement Disorder*" OR (TI "head bang*" OR AB "head bang*") OR (TI "tic disorder*" OR AB "tic disorder*") OR (TI "chronic motor tic disorder*" OR AB "chronic motor tic disorder*") OR (TI "vocal tic disorder*" OR AB "vocal tic disorder*") OR (TI "chronic tic disorder*" OR AB "chronic tic disorder*") OR (TI "Intellectual disabil*" OR AB "Intellectual disabil*") OR (TI "learning disabil*" OR AB "learning disabil*") OR (TI autism* OR AB autism*) OR (TI "developmental disabil*" OR AB "developmental disabil*") OR (DE "Learning Disorders" OR DE "Learning Disabilities" OR DE "Reading Disabilities") OR (TI IDD OR AB IDD) OR (TI "intellectual developmental disabil*" OR AB "intellectual developmental disabil*") OR (TI "IDD MH" OR AB "IDD MH") OR (TI "intellectual disabilit*" OR AB "intellectual disabilit*") OR (DE "Developmental Disabilities" OR DE "Specific Language Impairment") OR (TI "developmental disabilit*" OR AB "developmental disabilit*") OR (TI "developmental disorder*" OR AB "developmental disorder*")) AND (((TI "shared decision mak*" OR AB "shared decision mak*") OR (TI "decision aid*" OR AB "decision aid*") OR (TI "decision support*" OR AB "decision support*") OR (TI "decision making*" OR AB "decision making*") OR (TI "decision making intervention*" OR AB "decision making intervention*") OR (TI "decision making tool*" OR AB "decision making tool*") OR (TI SDM OR AB SDM) OR (TI "shared clinical decision*" OR AB "shared clinical decision*") OR (TI "shared medical decision*" OR AB "shared medical decision*") OR ((TI patient* OR AB patient*) AND (TI empower* OR AB empower*)) OR (DE "Decision Making" OR DE "Choice Behavior" OR DE "Group Decision Making" OR DE "Management Decision Making") OR (DE "Client Participation") OR (TI "patient participat*" OR AB "patient participat*") OR (DE "Decision Support Systems") OR (DE "Choice Behavior" OR DE "Autonomy" OR DE "Framing Effects" OR DE "Uncertainty") OR (TI "decision tree*" OR AB "decision tree*")

OR (TI "choice behavior*" OR AB "choice behavior*") OR (TI "Shared medical decision*" OR AB "Shared medical decision*") OR (TI "Shared treatment decision*" OR AB "Shared treatment decision*") OR (TI "Shared clinical decision*" OR AB "Shared clinical decision*")) OR ((((TI decision* OR AB decision*) OR (TI decid* OR AB decid*)) AND ((TI support* OR AB support*) OR (TI aid OR AB aid) OR (TI tool* OR AB tool*) OR (TI instrument* OR AB instrument*) OR (TI technique* OR AB technique*) OR (TI system* OR AB system*) OR (TI program* OR AB program*) OR (TI process* OR AB process*) OR (TI method* OR AB method*) OR (TI intervention* OR AB intervention*) OR (TI material* OR AB material*))))) AND ((DE "Parents" OR DE "Adoptive Parents" OR DE "Expectant Parents" OR DE "Fathers" OR DE "Foster Parents" OR DE "Homosexual Parents" OR DE "Mothers" OR DE "Parental Characteristics" OR DE "Single Parents" OR DE "Stepparents" OR DE "Surrogate Parents (Humans)") OR (TI parent* OR AB parent*))<br>Published Date: 19900101-20221231

Web of Science Core Collection (Clarivate Analytics) *from 01/01/1990 to 3/4/2022* (825 Results)

#1	TS=("mental disease" OR "mental diseas*" OR "neurodevelopment disorder*" OR "developmental delay*" OR "communication disorder*" OR "developmental communication disorder*" OR "childhood communication disorder*" OR "communicative dysfunction*" OR "communication disability*" OR "language disability" OR "language disabil*" OR "acquired language disorder*" OR "language disorder*" OR "speech sound disorder" OR "phonological disorder*" OR "speech sound disorder*" OR "fluency disorder" OR "childhood onset fluency disorder*" OR "fluency disorder*" OR stutter* OR "communication disorder" OR "social communication disorder*" OR "pragmatic communication disorder*" OR autism OR "autistic spectrum disorder*" OR "autism spectrum disorder*" OR autis* OR "attention deficit hyperactivity disorder" OR "attention deficit disorder*" OR hyperactivit* OR "adhd" OR "addh" OR "attention deficit hyperactivity disorder*" OR "hyperkinetic syndrom*" OR "learning disord*" OR "specific learning disorder*" OR "motor disorder*" OR "psychomotor disorder" OR "psychomotor disorder" OR "motor skills disorder*" OR "developmental coordination disorder*" OR "motor dysfunction" OR "motor dysfunction" OR "stereotypic movement disorder*" OR "movement disorder*" OR "head bang*" OR tic* OR "tic disorder*" OR "chronic motor tic disorder*" OR "vocal tic disorder*" OR "chronic tic disorder*" OR "intellectual disabil*" OR "learning disabil*" OR autism* OR "developmental disabil*" OR "intellectual impairment"

	OR "intellectual impairment" OR "learning disorder" OR "learning disorder" OR "idd" OR "intellectual developmental disabil*" OR "idd mh" OR "intellectual disabilit*" OR "developmental disorder" OR "developmental disabilit*" OR "developmental disorder*")
#2	TS=("shared decision mak*" OR "decision aid*" OR "decision support*" OR "decision making*" OR "decision making intervention*" OR "decision making tool*" OR "sdm" OR "patient participat*" OR "decision tree*" OR "choice behavior*" OR "shared medical decision*" OR "shared treatment decision*" OR "shared clinical decision*")
#3	(TS=(patient* NEAR empower*) OR TS=(decision* NEAR support) OR TS=(decision* NEAR aid*) OR TS=(decision* NEAR tool*) OR TS=(decision* NEAR instrument*) OR TS=(decision* NEAR technique*) OR TS=(decision* NEAR system*) OR TS=(decision* NEAR program*) OR TS=(decision* NEAR process*) OR TS=(decision* NEAR method*) OR TS=(decid* NEAR support) OR TS=(decid* NEAR aid*) OR TS=(decid* NEAR tool*) OR TS=(decid* NEAR instrument*) OR TS=(decid* NEAR technique*) OR TS=(decid* NEAR system*) OR TS=(decid* NEAR program*) OR TS=(decid* NEAR process*) OR TS=(decid* NEAR method*) OR TS=(decid* NEAR intervention*) OR TS=(decid* NEAR material*))
#4	#2 AND #3
#5	TS=(parent*)
#6	#1 AND #4 AND #5<br>Timespan: 1990-01-01 to 2022-03-04 (Publication Date)

Clinicaltrials.gov (https://clinicaltrials.gov/)

shared decision making | Neurodevelopmental Disorders OR communication disorders OR language disorders

WHO ICTRP (https://trialsearch.who.int/)

"shared decision makin*" AND "neurodevelopmental disorder*" OR "communication disorder*" OR "language disorder*"

Search Methods (second search)

Final search run on 07/07/2023 by *removed for a blind review* (initially created by *removed for a blind review*)

PubMed (NLM) *from 01/01/2022 to 7/7/2023* 115 results

(“communication disorder*”[tiab] OR “communicative disorder*”[tiab] OR “communication dysfunction*”[tiab] OR “communicative dysfunction*”[tiab] OR “communication disabilit*”[tiab] OR autis*[tiab] OR asperger*[tiab] OR “attention deficit”[tiab] OR ADHD[tiab] OR ADDH[tiab] OR “hyperkinetic syndrome*”[tiab] OR hyperactivity[tiab] OR “communication disorders”[mesh] OR “child development disorders, pervasive”[mesh] OR “attention deficit disorder with hyperactivity”[mesh])

AND

(("Decision Making, Shared"[Mesh] OR “shared decision mak*”[tiab] OR “decision aid*”[tiab] OR “decision support*”[tiab] OR “decision making*”[tiab] OR “decision making intervention*”[tiab] OR “decision making tool*”[tiab] OR “SDM”[tiab] OR “shared clinical decision*”[tiab] OR “shared medical decision*”[tiab] OR (patient*[tiab] AND empower*[tiab]) OR "Decision making"[MeSH] OR “decision support techniques”[Mesh] OR “patient participation”[MeSH] OR “patient participat*”[tiab] OR “decision support systems, clinical”[Mesh] OR “decision trees”[MeSH] OR "Choice Behavior"[Mesh] OR “decision tree*”[tiab] OR “choice behavior*”[tiab] OR “Shared treatment decision*”[tiab]) OR ((decision*[tiab] or decid*[tiab]) AND (support*[tiab] OR aid[tiab] OR tool*[tiab] OR instrument*[tiab] OR technique*[tiab] OR system*[tiab] OR program*[tiab] OR process*[tiab] OR method*[tiab] OR intervention*[tiab] OR material*[tiab])))

AND

(“Parents”[MeSH] OR parent*[tiab])

AND

(2022/01/01:3000/12/12[pdat])

Embase (Elsevier) *from 01/01/2022 to 7/7/2023* 120 results

(‘communication disorder*’:ab,ti OR ‘communicative disorder*’:ab,ti OR ‘communication dysfunction*’:ab,ti OR ‘communicative dysfunction*’:ab,ti OR autis*:ab,ti OR asperger*:ab,ti OR ‘attention deficit’:ab,ti OR adhd:ab,ti OR addh:ab,ti OR ‘hyperkinetic syndrome*’:ab,ti OR hyperactivity:ab,ti OR autism/exp OR ‘attention deficit hyperactivity disorder’/exp OR ‘communication disorder’/exp)

AND

'shared decision making'/exp OR 'shared decision mak*':ti,ab OR 'decision aid*':ti,ab OR 'decision support*':ti,ab OR 'decision making*':ti,ab OR 'decision making intervention*':ti,ab OR 'decision making tool*':ti,ab OR 'sdm':ti,ab OR (patient*:ti,ab AND empower*:ti,ab) OR 'decision making'/exp OR 'decision support system'/exp OR 'patient participation'/exp OR 'patient participat*':ti,ab OR 'clinical decision support system'/exp OR 'decision tree'/exp OR 'decision tree*':ti,ab OR 'choice behavior*':ti,ab OR 'shared medical decision*':ti,ab OR 'shared treatment decision*':ti,ab OR 'shared clinical decision*':ti,ab OR (decision* NEAR/3 support):ti,ab OR (decision* NEAR/3 aid*):ti,ab OR (decision* NEAR/3 tool*):ti,ab OR (decision* NEAR/3 instrument*):ti,ab OR (decision* NEAR/3 technique*):ti,ab OR (decision* NEAR/3 system*):ti,ab OR (decision* NEAR/3 program*):ti,ab OR (decision* NEAR/3 process*):ti,ab OR (decision* NEAR/3 method*):ti,ab OR (decid* NEAR/3 support):ti,ab OR (decid* NEAR/3 aid*):ti,ab OR (decid* NEAR/3 tool*):ti,ab OR (decid* NEAR/3 instrument*):ti,ab OR (decid* NEAR/3 technique*):ti,ab OR (decid* NEAR/3 system*):ti,ab OR (decid* NEAR/3 program*):ti,ab OR (decid* NEAR/3 process*):ti,ab OR (decid* NEAR/3 method*):ti,ab OR (decid* NEAR/3 intervention*):ti,ab OR (decid* NEAR/3 material*):ti,ab

AND

('parent'/exp OR parent*:ti,ab)

AND [2022-2023]/py

PsycInfo (EbscoHost) *from 01/01/2022 to 7/7/2023* 57 results

(TI “communication disorder*” OR “communicative disorder*” OR “communication dysfunction*” OR “communicative disfunction*” OR “communication disabil*” OR autis* OR asperger* OR “attention deficit” OR ADHD OR ADDH OR “hyperkinetic syndrome*” OR hyperactivity) OR (AB “communication disorder*” OR “communicative disorder*” OR “communication dysfunction*” OR “communicative disfunction*” OR “communication disabil*” OR autis* OR asperger* OR “attention deficit” OR ADHD OR ADDH OR “hyperkinetic syndrome*” OR hyperactivity) OR DE “autism spectrum disorders” OR DE “autistic traits” OR DE “attention deficit disorder with hyperactivity” OR DE “attention deficit disorder” OR DE “communication disorders” OR DE “language disorders” OR DE “social communication disorder”)

AND

((TI "shared decision mak*" OR AB "shared decision mak*") OR (TI "decision aid*" OR AB "decision aid*") OR (TI "decision support*" OR AB "decision support*") OR (TI "decision making*" OR AB "decision making*") OR (TI "decision making intervention*" OR AB "decision making intervention*") OR (TI "decision making tool*" OR AB "decision making tool*") OR (TI SDM OR AB SDM) OR (TI "shared clinical decision*" OR AB "shared clinical decision*") OR (TI "shared medical decision*" OR AB "shared medical decision*") OR ((TI patient* OR AB patient*) AND (TI empower* OR AB empower*)) OR (DE "Decision Making" OR DE "Choice Behavior" OR DE "Group Decision Making" OR DE "Management Decision Making") OR DE "Client Participation" OR (TI "patient participat*" OR AB "patient participat*") OR DE "Decision Support Systems" OR DE "Autonomy" OR DE "Framing Effects" OR DE "Uncertainty") OR (TI "decision tree*" OR AB "decision tree*") OR (TI "choice behavior*" OR AB "choice behavior*") OR (TI "Shared treatment decision*" OR AB "Shared treatment decision*")) OR ((TI decision* OR AB decision* OR TI decid* OR AB decid*) N3 (TI support* OR AB support* OR TI aid OR AB aid OR TI tool* OR AB tool* OR TI instrument* OR AB instrument* OR TI technique* OR AB technique* OR TI system* OR AB system* OR TI program* OR AB program* OR TI process* OR AB process* OR TI method* OR AB method* OR TI intervention* OR AB intervention* OR TI material* OR AB material*))

AND

((DE "Parents" OR DE "Adoptive Parents" OR DE "Expectant Parents" OR DE "Fathers" OR DE "Foster Parents" OR DE "Homosexual Parents" OR DE "Mothers" OR DE "Parental Characteristics" OR DE "Single Parents" OR DE "Stepparents" OR DE "Surrogate Parents (Humans)") OR (TI parent* OR AB parent*))

AND

DT 2022-2023

Web of Science Core Collection 85 results on 7/7/2023

#1	TS=(“communication disorder*” OR “communicative disorder*” OR “communication dysfunction*” OR “communicative disfunction*” OR “communication disabil*” OR autis* OR asperger* OR “attention deficit” OR ADHD OR ADDH OR “hyperkinetic syndrome*” OR hyperactivity)
#2	TS=("shared decision mak*" OR "decision aid*" OR "decision support*" OR "decision making*" OR "decision making intervention*" OR "decision making tool*" OR "sdm" OR "patient participat*" OR "decision tree*" OR "choice behavior*" OR "shared medical decision*" OR "shared treatment decision*" OR "shared clinical decision*")
#3	(TS=(patient* NEAR/3 empower*) OR TS=(decision* NEAR/3 support) OR TS=(decision* NEAR/3 aid*) OR TS=(decision* NEAR/3 tool*) OR TS=(decision* NEAR/3 instrument*) OR TS=(decision* NEAR/3 technique*) OR TS=(decision* NEAR/3 system*) OR TS=(decision* NEAR/3 program*) OR TS=(decision* NEAR/3 process*) OR TS=(decision* NEAR/3 method*) OR TS=(decid* NEAR/3 support) OR TS=(decid* NEAR/3 aid*) OR TS=(decid* NEAR/3 tool*) OR TS=(decid* NEAR/3 instrument*) OR TS=(decid* NEAR/3 technique*) OR TS=(decid* NEAR/3 system*) OR TS=(decid* NEAR/3 program*) OR TS=(decid* NEAR/3 process*) OR TS=(decid* NEAR/3 method*) OR TS=(decid* NEAR/3 intervention*) OR TS=(decid* NEAR/3 material*))
#4	#2 OR #3
#5	TS=(parent*)
#6	#1 AND #4 and #5
#7	PY=(2022-2023)
#8	#6 AND #7

Clinicaltrials.gov 9 results on 7/7/2023

Other terms: ("shared decision making") AND (autism OR "communication disorder*" OR "attention deficit" OR ADHD)

International Clinical Trials Registry Platform (World Health Organization, trialsearch.who.int) 4 results on 7/7/2023

Title: “shared decision making”<br><br>AND<br><br>Condition: autism OR "communication disorder*" OR "attention deficit" OR ADHD<br><br>Recruitment status: all
